# Impact of the spotted microarray preprocessing method on fold-change compression and variance stability

**DOI:** 10.1186/1471-2105-12-413

**Published:** 2011-10-25

**Authors:** Jérôme Ambroise, Bertrand Bearzatto, Annie Robert, Bernadette Govaerts, Benoît Macq, Jean-Luc Gala

**Affiliations:** 1 ICTEAM institute, ELEN department, Université Catholique de Louvain, Place du Levant 2, 1348 Louvain-la-Neuve, Belgium; 2 IREC institute, Center for Applied Molecular Technologies, Université Catholique de Louvain, Clos Chapelle-aux-Champs 30, 1200 Bruxelles, Belgium; 3 IREC institute, Epidemiology and Biostatistics department, Université Catholique de Louvain, Clos Chapelle-aux-Champs 30, 1200 Bruxelles, Belgium; 4 Institute of Statistics, Biotstatistics and Actuarial Science, Université Catholique de Louvain, 1348 Louvain-la-Neuve, Belgium

## Abstract

**Background:**

The standard approach for preprocessing spotted microarray data is to subtract the local background intensity from the spot foreground intensity, to perform a log2 transformation and to normalize the data with a global median or a lowess normalization. Although well motivated, standard approaches for background correction and for transformation have been widely criticized because they produce high variance at low intensities. Whereas various alternatives to the standard background correction methods and to log2 transformation were proposed, impacts of both successive preprocessing steps were not compared in an objective way.

**Results:**

In this study, we assessed the impact of eight preprocessing methods combining four background correction methods and two transformations (the log2 and the glog), by using data from the MAQC study. The current results indicate that most preprocessing methods produce fold-change compression at low intensities. Fold-change compression was minimized using the Standard and the Edwards background correction methods coupled with a log2 transformation. The drawback of both methods is a high variance at low intensities which consequently produced poor estimations of the p-values. On the other hand, effective stabilization of the variance as well as better estimations of the p-values were observed after the glog transformation.

**Conclusion:**

As both fold-change magnitudes and p-values are important in the context of microarray class comparison studies, we therefore recommend to combine the Edwards correction with a hybrid transformation method that uses the log2 transformation to estimate fold-change magnitudes and the glog transformation to estimate p-values.

## 1 Background

Gene expression microarray is a widely used technology in functional genomics that allows to measure efficiently the expression level of thousands of genes in a single experiment. Among the wide spectrum of available array technologies and suppliers, two common technologies are the in-situ oligonucleotide synthesised GeneChips developed by Affymetrix [1] and the spotted microarrays which are microscope slide spotted with a variable number of probes according to the biological application. Spotted microarrays use either cDNA as probe (Incyte Human UniGEM, Dualchip form Eppendorf, academic platforms,...) or oligonucleotide (Agilent gene expression Microarray, Applied Biosystems gene expression Microarray, Codelink Bioarray from GE Healthcare, NCI from Operon,...). The three major types of gene expression microarray applications are the class comparison, the class prediction and the class discovery [2]. In this paper, we focus on the preprocessing of spotted microarray data for a class comparison application where the goal is to identify differentially expressed genes between two conditions.

Whatever its application, the first analytical step in a spotted microarray experiment is the acquisition of an image file with an optical scanner. Then, the image analysis software segments the acquired image into spotted and unspotted regions and returns average and median of the pixels intensities for both the foreground and the surrounding area (named local background) of each spot. It is well known that the foreground intensity of a spot does not perfectly reflect the RNA abundance of its corresponding gene due to interferences of non-specific hybridization on the probe [3]. These interferences are named background noise and arise from many sources such as non-specific binding, deposit left due to incomplete washing, intrinsic fluorescence of the glass slides [4] or optical noise of the scanner. Han *et al*. showed that such interferences can be minimized by optimizing the numerous steps of the microarray experiment, and more particularly the hybridization and the washing steps [5]. The authors also showed that non-optimal protocols can lead to fold-change compression. In that context, de cremoux *et al*. discussed also the importance of pre-analytical steps for transcriptome analysis [6].

Raw data returned by the scanner have to be preprocessed in three successive steps [7]. The first step is the background correction for which the standard method implies to subtract an estimation of the background noise of a spot from the foreground intensity. The background noise is usually calculated as the mean of the pixels of its surrounding area and is named 'local background intensity'. The second step is the transformation of the corrected intensities for which the standard method consists in a log2 transformation. The third step is the normalization that is performed to calibrate the signal from different microarrays and to compare them together on an identical scale. Commonly used methods to normalize spotted microarray data either perform a global median normalization or a loess normalization [8].

The standard background correction method assumes that foreground intensities are affected additively by the background noise. Although well motivated, this standard method was widely criticized for several reasons. The best known drawback is that local background subtraction induces problems when foreground intensities are lower than local background intensities. Correction leads to negative corrected intensities and consequently to missing values after log2 transformation. Another cited drawback is the extreme variability of the log2 fold-changes obtained at low corrected intensities. To circumvent these drawbacks, alternatives to the standard background were proposed. [9-13]. Alternatively, the generalized logarithmic (glog) transformation was proposed as a valuable alternative to the log2 transformation [14,15] in order to stabilize the variance of low corrected intensities. The transformation is determined by the equation: $glog\left(x,\alpha ,\lambda \right)=log\left[x-\alpha +\sqrt{{\left(x-\alpha \right)}^{2}+\lambda }\right]$ where *α *and λ are two positive parameters. The glog transformation is sometimes referred as the generalized arcsinh transformation because of the relationship between the arcsinh and the log functions. $arcsinh\left(x\right)=log\left(x+\sqrt{x^{2}+1}\right)$. Methods were developed to estimate the parameters of the glog transformation [16,17]. Unlike the log2 transformation, the glog is defined for negative corrected intensities.

Eight distinct background correction methods were assessed for differential expression using data from two-color spotted cDNA microarrays by Ritchie *et al*. [18]. In this study, the variance stabilization method (VSN) of Huber *et al*. [15] was considered as a background correction method but was actually the combination of the Standard background correction method with an arcsinh transformation where parameters are computed to perform transformation and normalization in a single step. After the other background correction methods, a log2 transformation and a loess normalization were applied on the data before computing fold-changes with SAM regularized t-statistics and empirical Bayes moderated t-statistics. Using 9 Lucidea Universal ScoreCard (LUS) controls in a spike experiment, the authors also compared the average bias for each background correction method. Various transformation methods were compared by Cui *et al*. [7]. The glog transformation was recommended when low corrected intensities appear highly variable.

In this paper, we address the problem of the background correction and transformation of spotted microarray data and the subsequent impact on fold-change compression and on the variance of processed intensities. The first objective of this study was to compare various background correction methods and transformations commonly used in the literature. We propose to consider these two steps together because alternatives to the standard background correction methods as well as alternatives to the log2 transformation were initially proposed to circumvent the same problems: the high variability of low corrected intensities in the log2 scale and the missing values obtained after a log2 transformation of negative corrected intensities. These two successive preprocessing steps were assessed on datasets generated with two spotted microarray platforms (Duachip from Eppendorf and Codelink from GE Healthcare) as well as with a quantitative PCR platform (Taqman) from the MicroArray Quality Control (MAQC) project [19]. Data generated by the MAQC project provide a unique opportunity to assess the advantages and disadvantages of data analysis methods with the aim of reaching a consensus on microarray data analysis. Accordingly, data from the MAQC project were used previously in order to compare the third preprocessing step, i.e. the normalization [20]. A second objective of the study was to confirm the additive effect of the background noise on the foreground, the existence of which is the underlying hypothesis of the standard background correction method.

## 2 Methods

### 2.1 Comparison of Background correction and transformation methods

The first objective of this paper was to compare the effect of eight preprocessing methods combining four background correction methods and two transformations, on the processed intensities, on the log2 fold-changes and on p-values. Background correction methods are implemented in the backgroundCorrect function of the limma package which is a part of the Bioconductor project [21] developed in R. A short description of background correction methods and transformations appears below:

#### Standard

We refer to this method when background intensities are subtracted from foreground intensities.

#### No background

We refer to this method when the background intensities are not subtracted. The corrected intensities are thus equal to the foreground intensities. This method was recommended by other authors [3,22].

#### Edwards

In this method, the background intensities are subtracted if the difference between foreground and background is bigger than a pre-specified small threshold value. When the difference is smaller than this threshold value, subtraction is replaced by a smooth monotonic function [11].

#### Normexp

The Normexp method is based on the normal plus exponential convolution model [18]. The Normexp + offset method was not tested in this study because an offset is already artificially included to the method when it is coupled with the glog transformation (thanks to the *α *parameter).

#### log2 transformation

The log2 transformation is the most commonly used transformation for microarray data. This transformation stabilizes the data variance of high intensities but increases the variance at low intensities.

#### glog transformation

The glog transformation was individually developed by Durbin *et al*. [14] and Huber *et al*. [15] to stabilize the variance. The glog transformation and the estimation of *α *and λ parameters are implemented in the *transeS *and the *tranest *functions of the LMGene Bioconductor package [23]. To allow comparison with the log2 transformation, glog transformed intensities (which are in the natural log scale) were multiplied by log2(e). Practically, the glog transformation is equivalent to the regular logarithmic transformation at high intensities but close to a difference at low intensities.

### 2.2 Additive property of the background noise

The second objective of the paper was to confirm the additive property of the background on the foreground. This assumption which motivates the Standard background correction method was successfully tested on the Eppendorf data because this type of array contains three technical replicate spots used to measure gene expression levels. For each gene, three foreground intensities and three local background intensities are therefore available on a given array. The specific hybridization on each replicate spot for the same gene should be roughly constant. So, the observed differences between the three foreground intensities are mainly caused by the background noise. For each gene and for each Eppendorf array, the foreground and the background intensities of the three replicate spots were used to build a linear regression model using the foreground intensity as the response variable and the local background intensity as the predictor variable. If the assumption of additivity is true, an increase in the local background should produce the same increase in the foreground. As the Eppendorf array measures 294 genes, a total of 5 880 slopes (294 genes * 5 replicates * 2 samples * 2 sites) were obtained. The values of these slopes should consequently be close to 1 if the assumption of additivity holds.

## 3 Data

Data from the MAQC project were used in this study. As our study focuses on the preprocessing of spotted microarray data, we decided to perform analyses on a high density spotted oligonucleotide microarray platform (CodeLink Human Whole Genome from GE Healthcare) as well as on a low density spotted cDNA microarray platform (Dualchip Microarray from Eppendorf). As data from Site 2 suffer from annotation problems for both platforms, we only used data from sites 1 and 3 in this study. Among the samples measured in the MAQC study, sample A corresponds to the Universal Human Reference RNA (UHRR) from Stratagene while sample B corresponds to the Human Brain Reference RNA (HBRR) from Ambion. Samples C and D are two mixtures of the original samples. In order to maximize the range of the resulting fold-changes, we decided to use samples A and B (rather than C or D). Table 1 summarizes the data used in this study.

**Table 1:** Material of the MAQC project used in this study

Technology	Platform and Site	Sample A N Replicates	Sample B N Replicates	N Genes
Spotted oligo	GE Healthcare : Site 1	5	5	54 359
Spotted oligo	GE Healthcare : Site 3	5	5	54 359
Spotted cDNA	Eppendorf : Site 1	5	5	294
Spotted cDNA	Eppendorf : Site 3	5	5	294
Quantitative PCR	TaqMan	4	4	1 004

Data acquired with the GE Healthcare platform from sites 1 and 3 were downloaded from the Gene Expression Omnibus (GEO) repository (GEO accession: GSE5350) [24]. Raw data were imported in R using the *codelink *package and were preprocessed using the eight different methods. Data were then normalized between samples A and B using a global median normalization. Finally, as the number of biological replicates is relatively low [25], the *eBayes *algorithm [26] of the *limma *Bioconducor package was used to compute the log2 fold-changes and p-values of the 54 359 genes between samples A and B.

Data acquired with the Eppendorf platform from sites 1 and 3 were downloaded from the GEO repository. Raw data were imported in R and preprocessed using the eight different methods. For this platform, data acquired at low, medium and high photomultiplier tube (PMT) voltage were available. In this study, we only considered data acquired at low PMT Voltage in order to avoid saturation problems. Processed data corresponding to samples A and B were normalized using internal standards and housekeeping genes, as recommended by the manufacturer. As Eppendorf platforms contain three replicate spots to measure the level of expression of a single gene, the average of these replicate spots was computed for each gene. Linear models of the *limma *Bioconducor package were used to compute fold-changes and p-values for the 294 genes between samples A and B.

Normalized data from the Taqman quantitative PCR were downloaded from the GEO repository and linear models of the *limma *package were used to compute fold-changes and p-values for the 1 004 genes between samples A and B. In this study, these values are referred as gold-standard fold-changes and gold-standard p-values.

## 4 Results and discussion

### 4.1 Comparison of background correction methods

#### 4.1.1 Fold-change compression

Product-moment correlation (r) and intraclass correlation (ICC) coefficients were computed between the log2 gold-standard fold-changes and those obtained from both platforms (Eppendorf and GE Healthcare), from both sites (Sites 1 and 3) after each preprocessing method (Table 2). The product-moment correlation coefficient is a measure of the strength of linear dependence between two variables while the intraclass correlation coefficient is designed to assess consistency or conformity between two or more quantitative measurements [27]. Considering the GE Healthcare platform, correlation coefficients were computed using the 856 genes which are commonly measured on the GE Healthcare Codelink Microarray and on the quantitative PCR. Regarding the Eppendorf platform, correlation coefficients were computed using the 132 genes which are commonly measured on the Eppendorf Dualchip and on the Taqman quantitative PCR.

**Table 2:** Correlation between Microarray and Taqman fold-changes

**Transformation Background cor**.	GEH S1 r - ICC	GEH S3 r - ICC	EPP S1 r - ICC	EPP S3 r - ICC
Log2				
Standard	**0.86 - 0.78**	**0.86 - 0.81**	**0.84 - 0.72**	**0.82 - 0.68**
No background	0.84 - 0.66	0.84 - 0.68	0.68 - 0.30	0.67 - 0.27
Edwards	**0.86 - 0.78**	**0.86 - 0.82**	**0.84 - 0.72**	**0.82 - 0.68**
NormExp	0.86 - 0.77	0.86 - 0.79	0.83 - 0.67	0.81 - 0.63
Glog				
Standard	0.83 - 0.68	0.83 - 0.70	0.77 - 0.55	0.76 - 0.53
No background	0.81 - 0.60	0.81 - 0.63	0.65 - 0.33	0.65 - 0.30
Edwards	0.83 - 0.68	0.83 - 0.70	0.79 - 0.61	0.77 - 0.59
NormExp	0.83 - 0.67	0.83 - 0.69	0.77 - 0.54	0.77 - 0.61

Product-moment correlation (r) and intraclass Correlation (ICC) between log2 fold-changes obtained with quantitative PCR and log2 fold-changes obtained with microarray.

**Figure 1:**
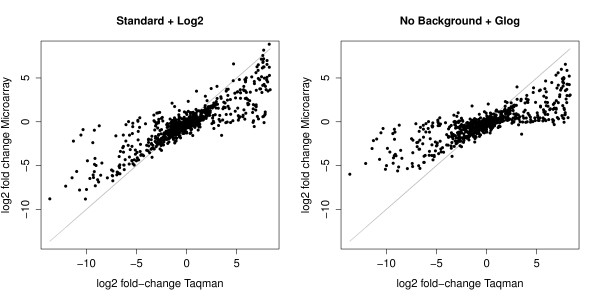
**Scatter plot between microarray and Taqman**. Scatter plot of log2 fold-changes obtained from quantitative PCR versus the log2 fold-changes obtained from microarray after the best- (Standard background correction with log2 transformation) and the worst- (No background correction with glog transformation) preprocessing methods. Microarray data leads to fold-change compressions for many genes, especially with the No background correction and the glog transformation (see Figure2).

The Standard and the Edwards correction methods followed by a log2 transformation showed the highest correlations with both platforms and sites. The No background correction method generated poor correlation coefficients, especially when combined with the glog transformation. A scatter plot of the log2 fold-changes obtained with the quantitative PCR and those obtained with the GE Healthcare in S1 (Figure 1) was produced using the best- (Standard background correction with log2 transformation) and the worst- (No background correction with glog transformation) preprocessing methods.

As illustrated in Figure 1, microarray data leads to fold-change compressions for many genes when compared to fold-changes derived from quantitative PCR. This compression effect was studied with the eight preprocessing methods and with both microarray platforms. Absolute values of the log2 fold-changes obtained with microarray data were computed after each preprocessing method. These absolute values were subtracted from the absolute values of gold-standard log2 fold-changes to obtain the fold-change compressions (in the log2 scale). Fold-change compressions obtained for each gene were used to construct a lowess curve representing the average fold-change compression as a function of the average processed intensity for each preprocessing method. The average processed intensities on the x-axis of the lowess curve were computed for each gene as the minimum of average intensities in sample A and B after the Standard background correction and the log2 transformation. The x-axis is therefore also in the log2 scale.

**Figure 2:**
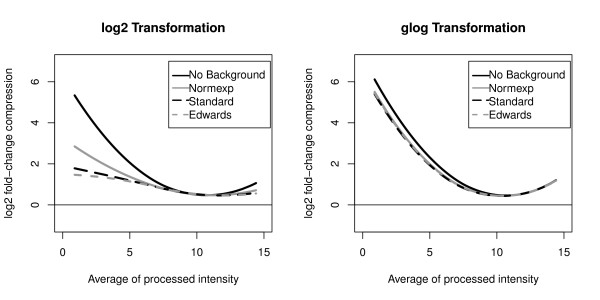
**GE Healthcare Fold-change compression**. Lowess curves of the log2 fold-change compression estimated with the GE Healtchare platform as a function of the average processed intensity. The No background correction as well as the glog transformation produce high fold-change compression at low processed intensities. The fold-change compression at low intensities affects the correlation between gold-standard fold-changes and those obtained with microarray data (see Table 2).

Considering the GE Healthcare platform, the 856 genes in both sites were used to compute 1712 fold-change compressions and to construct a lowess curve for each background correction and for both transformations (Figure 2). When data are log2 transformed, the average fold-change compression depends greatly on the background correction methods. When the No background method is applied, the average fold-change compression is higher than 5 at low intensities. In these conditions, when a fold-change of 64 is obtained from quantitative PCR, the average fold-change obtained from microarray data with the No background method is therefore close to $2\;\;\left(2=\frac{64}{2^{5}}\right)$. When either Standard or the Edwards method are chosen, fold-change compression is lower than 2 at low intensities. At higher intensity, the average fold-change compression is close to 0 regardless of the background correction and transformation method.

**Figure 3:**
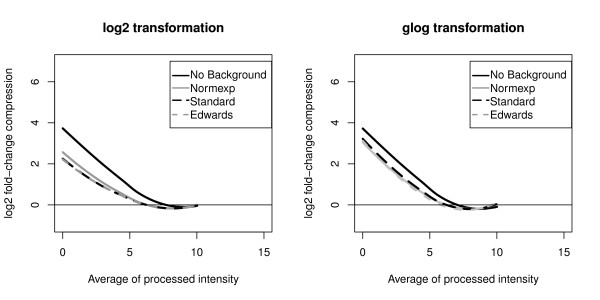
**Eppendorf Fold-change compression**. Lowess curves of the log2 fold-change compression estimated with the Eppendorf platform as a function of the average processed intensity. Fold-change compression is minimized when the Standard and the Edwards background correction methods are used with a log2 transformation as with the GE Healthcare platform (see Figure 2).

Considering the Eppendorf platform, the 132 genes in both sites were used to compute 264 fold-change compressions and to construct a lowess curve for each background correction and for both transformations (Figure 3). Profiles of the lowess curves are close to those obtained with the GE Healthcare platform. At low intensities, the average fold-change compression is minimized when using either Standard or Edwards correction method with a log2 transformation.

#### 4.1.2 Variance of the processed intensities

**Figure 4:**
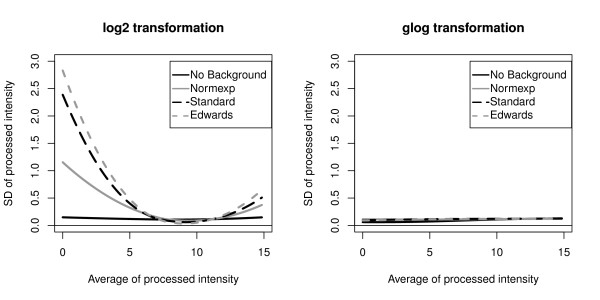
**GE Healthcare standard deviation**. Lowess curves of the standard deviation (SD) of the GE Healthcare processed data as a function of the average processed intensity

The intensity variances were computed with the eight preprocessing methods and with both microarray platforms. Regarding the GE Healtcare platform, data were preprocessed using the eight different methods. The 54 359 genes assessed on both samples and both sites in the 5 replicate arrays were used to compute a total of 217 436 (54 359 genes * 2 conditions * 2 sites) standard deviations for each preprocessing method. These standard deviations were used to construct a lowess curve representing the intensity standard deviation as a function of the intensity average (Figure 4). Average processed intensities on the x-axis of these lowess curves were computed as the average of the processed intensities for each gene in the 5 replicate arrays after a Standard background correction and a log2 transformation. The x-axis is therefore also in the log2 scale. The Standard, the Edwards and the Normexp background correction methods produced high average standard deviation at low processed intensities. Variance was effectively stabilized with the No background correction method as well as with the glog transformation.

**Figure 5:**
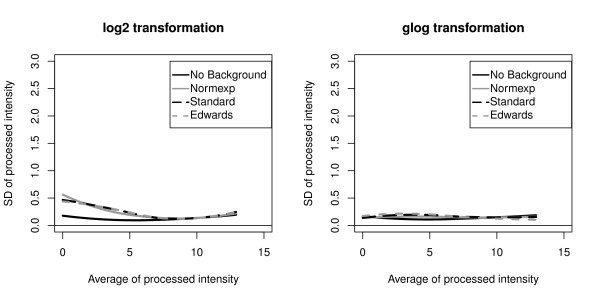
**Eppendorf Healthcare standard deviation**. Lowess curve of the standard deviation (SD) of Eppendorf processed data as a function of the average processed intensity

Regarding the Eppendorf platform, the 294 genes measured on both samples and both sites in 5 replicate arrays were used to compute a total of 1 176 variances (294 genes * 2 conditions * 2 sites) for each preprocessing method. 1 176 average processed intensities were computed with the Standard background correction method followed by the log2 transformation. The lowess curves constructed from the variances obtained after each preprocessing method are shown in Figure 5. The highest variances were obtained when the Standard, the Edwards and the Normexp background correction methods were combined with a Log2 transformation, an effect which was also observed with the GE Healthcare technology. Compared to the later, the maximal variances obtained with Eppendorf were nevertheless much smaller. A potential explanation is that gene expression levels in Eppendorf Dualchip are estimated as the average of three technical replicate spots.

As variance stabilization is a step in the preprocessing of microarray data that can greatly benefit the performance of subsequent statistical modeling and inference [28], we assessed its impact on p-values. Product-moment and intraclass correlation coefficients were computed between the cumulative Gaussian quantiles of the gold standard p-values and those obtained from both platforms and both sites after each of the eight preprocessing methods (Table 3). Compared to the log2 transformation, the glog transformation which effectively stabilizes the variance (Figure 4, 5), produced generally higher intraclass correlation and comparable product-moment correlation.

**Table 3:** Correlation between cumulative Gaussian quantiles of p-values obtained with Taqman and Microarray

**Transformation Background cor**.	GEH S1 r - ICC	GEH S3 r - ICC	EPP S1 r - ICC	EPP S3 r - ICC
Log2				
Standard	0.50 - 0.28	0.49 - 0.36	0.41 - 0.15	0.47 - 0.19
No background	0.52 - 0.45	0.51 - 0.48	0.49 - 0.34	0.48 - 0.24
Edwards	0.50 - 0.27	0.48 - 0.34	0.42 - 0.16	0.49 - 0.22
NormExp	0.50 - 0.30	0.50 - 0.39	0.40 - 0.15	0.48 - 0.14
Glog				
Standard	0.52 - 0.46	0.51 - 0.47	0.47 - 0.35	0.51 - 0.36
No background	0.50 - 0.43	0.49 - 0.47	0.47 - 0.34	0.45 - 0.19
Edwards	0.52 - 0.45	0.51 - 0.47	0.45 - 0.32	0.51 - 0.34
NormExp	0.52 - 0.45	0.50 - 0.47	0.48 - 0.38	0.50 - 0.27

Product-moment correlation (r) and intraclass Correlation (ICC) between the cumulative Gaussian quantiles of p-values obtained with quantitative PCR and cumulative Gaussian quantiles of p-values obtained with microarray.

#### 4.1.3 Evaluation of a hybrid transformation method

Results presented in previous sections showed that the combination of the Edwards method with log2 transformation produced low fold-change compression but led to poorer p-values estimations. Conversely, the combination of Edwards method with the glog transformation produced high fold-change compression at low processed intensities but led to better p-values estimations. When microarrays are used in a class comparison application, both fold-change magnitudes and p-values are considered. We propose therefore to combine the Edwards correction with a hybrid transformation method that uses the log2 transformation to estimate fold-change magnitudes and the glog transformation to estimate p-values.

**Figure 6:**
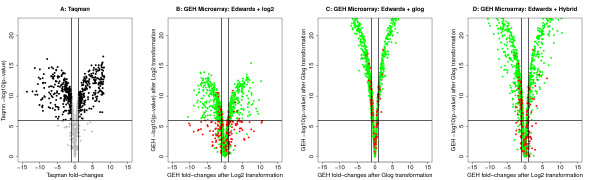
**Volcano plots**. (A): Volcano plot corresponding to the Taqman quantitative PCR. Black points in both upper corners correspond to differentially expressed genes (log2 fold-change higher than 1 and p-value lower than 10-6); (B): Volcano plot of the GE Healthcare data with the Edwards background correction method and a log2 transformation. A high number of false negatives (red points) are produced because of poor estimations of p-values; (C): Volcano plot of the GE Healthcare data with the Edwards background correction method and a glog transformation. The glog transformation compresses the fold-changes and produces a high number of false negatives; (D): Volcano plot of the GE Healthcare data with the Edwards background correction method and a hybrid transformation. Fold-changes and p-values are estimated using the log2 and the glog-transformations, respectively.

In this context, volcano plots [25] were used to compare this hybrid transformation approach with log2 and glog transformations on 1712 genes (856 genes * 2 sites) that are commonly measured by GE Healthcare Microarray and Taqman quantitative PCR. Differentially expressed genes correspond to the set of points lying in the upper corner of the volcano plot corresponding to Taqman quantitative PCR (Figure 6A). Genes corresponding to these points have indeed an absolute log2 fold-change higher than 1 and a p-value lower than 10^-6^. This p-value threshold was fixed by taking into account multiple testing. Three volcano plots were generated from GEH microarray data (Figures 6B-C-D). True positives and true negatives were plotted in green while false positives and false negatives were plotted in red.

The first volcano plot (Figure 6B) is depicted using fold-changes and p-values obtained after the Edwards background correction method and the log2 transformation. This preprocessing method produced p-values that were lower than those obtained with quantitative PCR, generating consequently a high number of false negatives (table 4). The second volcano (Figure 6C) plot is depicted using fold-changes and p-values obtained after the glog transformation. The glog transformation produced lower p-values (i.e. higher -log10(p-values)) than the log2 transformation but resulted in fold-change compression. Finally, the hybrid method that used the log2 transformation for fold-change estimations and the glog transformation for p-value estimations (Figure 6D) produced slightly more false positives but less false negatives. Specificity and sensitivity were computed for each transformation method. Compared to the log2 and the glog transformations, the hybrid method produced higher sensitivity but lower specificity. Considering the classification accuracy, the highest value was obtained with the hybrid method (0.756).

**Table 4:** Comparison of the transformation methods

	Edwards + Log2	Edwards + glog	Edwards + hybrid
TRUE POSITIVES	585	643	698
TRUE NEGATIVES	636	630	597
FALSE POSITIVES	81	87	120
FALSE NEGATIVES	410	352	297

SENSITIVITY	0.588	0.646	0.702
SPECIFICITY	0.887	0.879	0.833

CLASSIFICATION ACCURACY	0.713	0.744	0.756

Number of true positives, true negatives, false positives and false negatives generated by microarray data with an Edwards background correction method combined with three transformations methods.

### 4.2 Additive property of the background noise

**Figure 7:**
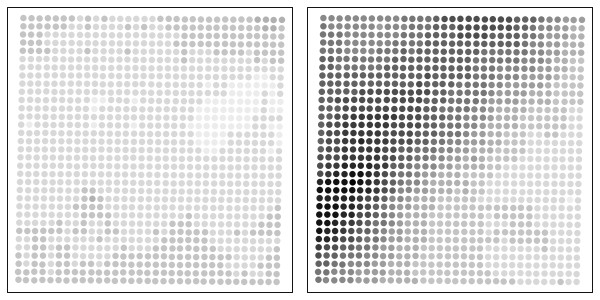
**Local background spatial plot**. Spatial plot of the local background intensities of the two Eppendorf arrays. The local background intensity seems to depend strictly on the spatial localization

Before assessing the additive property of the background noise on the foreground intensity, it is important to consider the potential effect of the foreground intensity on the local background intensity. For this purpose, spatial plots of the local background intensity were constructed with both arrays of a Dualchip. The spatial plots represent the local background intensities as a function of their spatial positions and are displayed in Figure 7. Dark points correspond to high local background intensities and consequently to high background noise intensities. As seen in Figure 7, the local background intensity seems to depend strictly on the spatial localization. It can therefore be inferred that the local background intensities are not influenced by the corresponding foreground intensities.

For each gene and for each Eppendorf array, the foreground and the background intensities of the three replicated spots were used to build a linear regression model. The additive property of background noise on the foreground was tested by computing the slopes of the 5 880 linear regression models. A robust estimation of the average slope (1.22) and its 95% confidence interval (0.94 ; 1.49) were obtained by trimming 5 % of the 5 880 slopes. On average, the foreground intensity of a spot with a fixed specific hybridization increases by 1.22 unit when its local background intensity increases by 1 unit. As we showed previously, local background intensities depend on the spatial localization and are independent of their corresponding foreground intensities. It can be inferred therefore that the background noise has an additive effect on foreground intensities.

## 5 Conclusion

In this study, we addressed the problem of background correction and transformation in spotted microarray data. We compared features of eight preprocessing methods which combine four background correction and two transformation methods.

We first compared the correlations between gold-standard fold-changes obtained from quantitative PCR and fold-changes obtained from microarray data. The best correlations were obtained with the Edwards and the Standard background corrections coupled with the log2 transformation. The lowest correlations were obtained with the No background correction method and with all preprocessing using the glog transformation. These results were explained by plotting lowess curves of the fold-change compression as a function of the average processed intensity. While all preprocessing methods produced low fold-change compression at high processed intensity, the different preprocessing methods differed markedly in terms of fold-change compression at low processed intensities. Accordingly, the fold-change compression was minimized using either the Standard or the Edwards background correction methods with the log2 transformation. Using a glog transformation conducted to high fold-change compression whatever the background correction method. It is of note that product-moment correlation coefficients are affected by the fold-change compression because this effect is highly dependent on the average processed intensity. A constant fold-change compression across the whole range of processed intensities would indeed have an impact on intraclass correlation coefficients but no impact on the product-moment correlation coefficients.

These results provide information that are complementary to those published in previous studies which reported that microarray data exhibit fold-change compression [5,18,29]. While the study of Han *et al*. focuses on the protocol technical aspects that can improve the signal-to-noise ratio and decrease the fold-change compression, our study rather focuses on the best choice of the data preprocessing method. While average biases were only estimated on 9 LUS control probes in the Ritchie's study, 856 and 132 probes were used in our study to compute fold-change compression on the GE Healthcare and on the Eppendorf platforms, respectively. Moreover, in Ritchie's study, the compression factors were only available for 2 of the 9 available LUS control probes for which most background correction methods produced a fold-change compression but the VSN method (equivalent to Standard background correction plus glog transformation) surprisingly produced a fold-change expansion. In the current study, all preprocessing methods produced fold-change compression. Furthermore the compression affected mainly low intensity data, an effect that can be minimized by using a combination of the Standard or Edwards background correction with a log2 transformation. The observed differences between these current results and those of Ritchie *et al*. could be explained by inherent differences between both datasets and by real observed technical differences between one- and two-color microarray readings [30]. In one-color arrays, the background noise caused by non-specific hybridization and deposits may differ for both the target and control spots used to quantify the expression fold-change. In two-color microarrays, the control and target samples are both hybridized on the same array. The signal due to non-specific hybridization and deposits are consequently more alike.

In microarray class comparison studies, effect sizes and p-values are computed by dividing the log2 fold-changes by an estimate of variability. The combination of Edwards (or Standard) method with the log2 transformation produced low fold-change compression but extremely high variance at low processed intensities. At the opposite, the combination of Edwards (or Standard) method with the glog transformation produced high fold-change compression but good variance stabilization at low processed intensities. The impact of the fold-change compression and variance stabilization on the p-values estimation was assessed by computing the correlations between the cumulative Gaussian quantiles of the gold standard p-values obtained from quantitative PCR and the cumulative Gaussian quantiles of p-values obtained from microarray data. Compared to the log2 transformation, the glog transformation which effectively stabilizes the variance across the whole range of processed intensities, produced generally higher intraclass correlation and comparable product-moment correlation. These results are in line with those obtained by Ritchie *et al*. which showed that the best performing methods are those stabilizing the variance for the purpose of detecting differential expression. These results also agree with those of Cui *et al*. [7] which stated that stabilizing the variance of log2 fold-changes is important for statistical inferences that assume constant variance across the experiment. While the No background correction is sometimes recommended in the literature [3,22] because it decreases variance at low processed intensities, our results show that the combination of the Edwards or Standard background correction with a glog transformation represents a better alternative for the p-values computation. Furthermore, we also recommend subtraction of the background as we have confirmed the additive property of the background noise on foreground intensity values in this study.

When microarrays are used in a class comparison application, both fold-change magnitudes and p-values are considered. Historically, the first method to identify differentially expressed genes was based on the fold-change [2,29]. A change of a least two-fold (up or down) was generally considered meaningful. Because this method did not take into account the variance of gene expression, it was replaced by statistical inference methods and p-values. P-values are nowadays used to rank the gene according to the more probable differential expression. Nevertheless, fold-change remains an important feature because it is generally accepted that the greater the magnitude of change, the higher the likelihood of physiologic or pathologic significance [29]. In the context of class comparison, we therefore recommend to combine the Edwards correction with a hybrid transformation method that uses the log2 transformation to estimate fold-change magnitudes and the glog transformation to estimate p-values. This hybrid method was compared to the log2 and to the glog transformation and was found to lead to the lowest number of incorrect decisions. Although comparable to the Standard method, the Edwards method is preferable because it avoids the occurrence of missing values even when combined with a log2 transformation. Moreover, when microarrays are used in the context of class prediction, the most important feature is the stability of the variance across the whole range of processed intensities. In this context, Parson *et al*. [31] have indeed showed that stabilizing the variance can improve the classification accuracy. We therefore recommend to use Standard or Edwards background correction with a glog transformation in order to stabilize the variance in this kind of microarray application. As shown here, the choice of the preprocessing steps should therefore not only be based on the type of microarray platforms but also defined according to the type of application.

## Authors' contributions

JA performed the microarray data analysis presented in this the paper and participated in compilation of the publication. AR and BG supervised the statistical analysis component of the work and assisted with review of the manuscript. JLG, BM and BB supervised the work and assisted in finalization of the completed manuscript. All authors read and approved the final manuscript.
